# “The Great Mimicker”: An Unusual Etiology of Cytopenia, Diffuse Lymphadenopathy, and Massive Splenomegaly

**DOI:** 10.1155/2015/637965

**Published:** 2015-10-22

**Authors:** Mazen Zaarour, Chanudi Weerasinghe, Elias Moussaly, Shafinaz Hussein, Jean-Paul Atallah

**Affiliations:** ^1^Department of Medicine, Staten Island University Hospital, North Shore-LIJ Health System, Staten Island, New York, NY 10305, USA; ^2^Department of Pathology, Staten Island University Hospital, North Shore-LIJ Health System, Staten Island, New York, NY 10305, USA; ^3^Division of Hematology and Oncology, Department of Medicine, Staten Island University Hospital, North Shore-LIJ Health System, Staten Island, New York, NY 10305, USA

## Abstract

Sarcoidosis is an idiopathic multisystem disease characterized by the formation of noncaseating granulomas. It frequently presents with pulmonary infiltrates and bilateral hilar and mediastinal lymphadenopathy. Splenic involvement is common, but massive splenomegaly is a rare occurrence. Sarcoidosis is known as “the great mimicker” (or “the great imitator”) since it exhibits a myriad of symptoms, mimicking other inflammatory, infectious, and neoplastic conditions, including lymphoma. Herein, we report the case of a 44-year-old male patient who was found to have bicytopenia, hypercalcemia, diffuse lymphadenopathy, and massive splenomegaly, a constellation of findings suggestive of underlying lymphoma. Interestingly, lymph node biopsy showed noncaseating granulomas suggestive of sarcoidosis, without evidence of malignancy.

## 1. Introduction

Sarcoidosis is a chronic inflammatory disorder of unknown origin which occurs mainly in young people [1, 2]. It is characterized by the presence of noncaseating granulomas. Although the lung is the most common organ involved, the disease can affect any organ, including the spleen [1]. Granulomatous infiltration of the spleen is common in sarcoidosis and is often asymptomatic [2]. Splenomegaly is unusual, and massive splenomegaly is very rare [3].

The symptoms of sarcoidosis, if present, are nonspecific. The presence of noncaseating granulomas is also not pathognomonic of the disease, as it can be seen in malignancy [1]. Moreover, the involvement of the reticuloendothelial system in sarcoidosis, as evidenced by enlarged lymph nodes and splenomegaly, often mandates tissue examination to exclude an underlying masked lymphoma. Sarcoidosis is well known to be “the great mimicker” (or “the great imitator”), since it exhibits a myriad of symptoms, mimicking other inflammatory, infectious, and neoplastic conditions, including lymphoma.

Herein, we report the case of a 44-year-old male patient who was found to have bicytopenia, hypercalcemia, diffuse lymphadenopathy, and massive splenomegaly, a constellation of findings suggestive of underlying lymphoma. Surprisingly, lymph node biopsy showed noncaseating granulomas suggestive of sarcoidosis, without evidence of malignancy.

## 2. Case Presentation

We report the case of a 44-year-old Caucasian male who was referred to our hospital by his primary physician for abnormal outpatient laboratory test values. The patient had been healthy until 5 months prior to admission, when he started to have progressively worsening fatigue. Outpatient blood tests revealed kidney injury, hypercalcemia, and anemia, findings that required hospitalization.

On the day of admission, the patient's only complaint was severe fatigue. Upon further questioning, he admitted having a 70-pound unintentional weight loss over the last 18 months. He denied any fever, chills, night sweats, cough, rash, or joint or abdominal pain. His prior medical history consisted of diabetes mellitus, gout, and hyperlipidemia. The patient was a nonsmoker and had no allergies. His family history was noncontributory.

On physical exam, the patient's body temperature was 98.6°F, blood pressure was 159/92 mmHg, and heart rate was 100/min. Cardiovascular and pulmonary exams were unremarkable. Left upper quadrant tenderness was noted on the abdominal exam, as well as a firm and enlarged spleen, which was palpable below the umbilicus. No rash, cervical, or axillary lymphadenopathy was identified.

**Figure 1:**
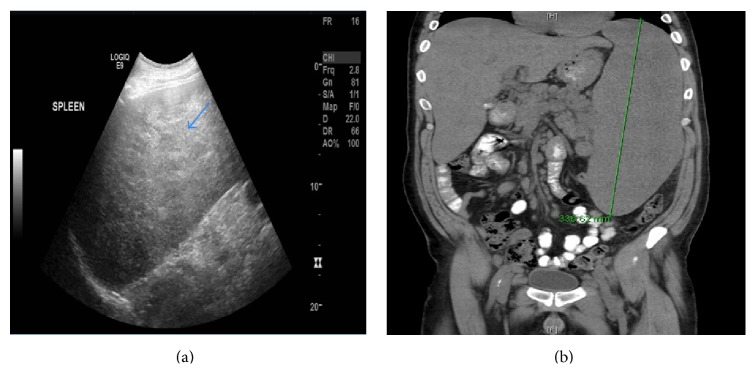
Massive splenomegaly. (a) Sagittal sonographic view of the spleen showing a markedly enlarged and diffuse heterogeneous spleen (blue arrow) measuring 30 cm in length. (b) Coronal noncontrast CT of the abdomen and pelvis showing enlarged spleen reaching 33.6 cm in length.

**Figure 2:**
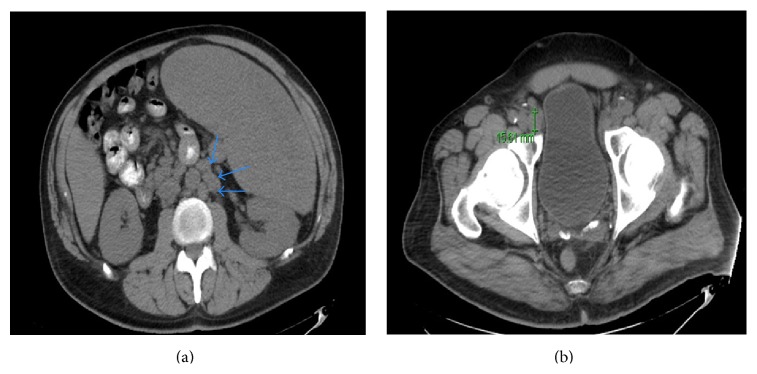
Diffuse lymphadenopathy. (a) Transverse CT of the abdomen showing enlarged para-aortic lymph nodes (blue arrows) reaching 14 mm in the shortest axis. (b) Transverse CT of the pelvis showing enlarged inguinal lymph nodes reaching 15 mm in the shortest axis.

Laboratory analysis showed a normocytic anemia with a hemoglobin of 6.7 g/dL, a hematocrit of 21.4%, and a mean corpuscular volume (MCV) of 82.3 *μ*m^3^. The rest of the hematologic panel was as follows: white blood cell count of 3.93 × 10^9^/L, platelet count of 254 × 10^9^/L, and erythrocyte sedimentation rate (ESR) of 93 mm/h. A peripheral blood smear was within normal limits. In addition, hypercalcemia of 13.7 mg/dL was noted, along with a blood urea nitrogen (BUN) of 33 mg/dL and a serum creatinine of 2.39 mg/dL, findings consistent with kidney injury. The renal function was normal three years ago. Liver enzymes were normal. An abdominal sonogram showed a markedly enlarged and diffusely heterogeneous spleen measuring 30 cm in length (Figure 1(a)). Enlarged kidneys with normal echogenicity were found as well (right kidney 13 cm, left kidney 15.5 cm). A noncontrast computed tomography (CT) scan of the abdomen and pelvis confirmed the presence of massive splenomegaly (Figure 1(b)), along with multiple mildly enlarged paraaortic, mesenteric, and bilateral iliac chain lymph nodes (Figure 2). A chest radiograph was unremarkable; however, a CT chest revealed diffuse mediastinal, lower cervical, and axillary adenopathy.

The patient received packed red blood cells transfusions to maintain his hemoglobin level around 8 mg/dL. He was also given intravenous fluids to treat the hypercalcemia and received one dose of pamidronate, which helped to lower the calcium level to as low as 11 mg/dL over the next few days.

**Figure 3:**
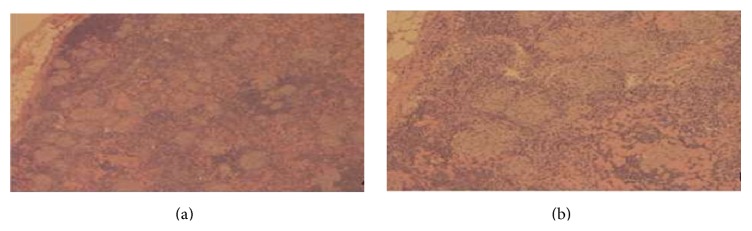
Nonnecrotizing granulomata. Low- (a) and high-magnification (b) photomicrograph of a section from an inguinal lymph node. It shows that the lymph node is replaced by numerous small compact nonnecrotizing granulomata.

The presence of massive splenomegaly and diffuse lymph node enlargement was concerning lymphoma. Further workup showed anemia of chronic disease and elevated vitamin D 1,25(OH)_2_ levels (with low PTH levels). Anti-nuclear antibody, HIV test, monospot test, and purified protein derivative (PPD) were negative. Additional studies are listed in Table 1. A bone marrow biopsy revealed a hypercellular marrow with negative cultures and cytogenetic analysis. PCR analyses for Bcr/Abl and JAK2 mutation were both negative. An excisional biopsy of a left inguinal lymph node showed that the lymph node was extensively involved with small compact nonnecrotizing granulomata (Figure 3). Gomori methenamine silver (GMS) and acid-fast bacillus (AFB) staining showed no fungal organisms or acid-fast organisms, respectively. There was no morphologic or immunophenotypic evidence of malignancy. Additional serum studies showed an angiotensin-converting enzyme (ACE) level of 73 U/L (reference range 9–67). These findings led to a diagnosis of sarcoidosis, for which the patient was started on prednisone 40 mg/day and discharged home few days later.

Four weeks after the initiation of therapy, the patient's calcium level was 10.3 mg/dL, along with a hemoglobin of 9.4 g/dL and a creatinine of 1.38 mg/dL. 2 weeks later, ACE level was 31 mg/dL. 8 weeks after hospital discharge, a fluorine-18 fluorodeoxyglucose (FDG) PET imaging, done while the patient was still on treatment, showed no focal FDG avid lesions, along with stable non-FDG avid thoracic and abdominal adenopathy. The patient continued to be in good health 9 months after his diagnosis and had shown no progression of sarcoidosis.

## 3. Discussion

Sarcoidosis is a chronic idiopathic granulomatous disease which can affect all age groups [1]. It has a slight predilection for women in the third to fifth decades of life. Sarcoidosis can affect virtually any organ system, with the lungs and mediastinal lymphatic system being affected in up to 90% of patients. In this setting, bilateral hilar adenopathy, with or without interstitial lung disease, is a common finding. Extrathoracic sarcoidosis is also common, with liver and spleen involvement described in half of autopsy cases [2, 3]. Other commonly involved organs are the skin, the joints, and the eyes.

Sarcoidosis can be asymptomatic in some patients. If present, symptoms are both systemic (fever, weight loss, and fatigue) and/or organ-specific (shortness of breath, chest pain, and cough) [1]. There is no single laboratory test for the diagnosis. However, cytopenia, eosinophilia, and hypergammaglobulinemia are common findings. Hypercalcemia and/or hypercalciuria are also found in some cases. ACE, produced by the epithelial cells of granulomas, is detected in the serum of 60% of patients; however, its value in diagnosing ormanaging sarcoidosis remains controversial [4]. Soluble interleukin-2 receptor (sIL-2R) concentration, a marker of T-cell activation, is considered to reflect disease activity. A biopsy from the involved organ that is most easily accessed is recommended and is the only way to establish the diagnosis [4, 5].

The diagnosis of sarcoidosis is based on criteria from the American Thoracic Society (ATS), the European Respiratory Society (ERS), and the World Association of Sarcoidosis and Other Granulomatous Disorders (WASOG) [6]. These criteria include the following: the presence of clinicoradiological findings suggestive of sarcoidosis, the presence of histological evidence of noncaseating epithelioid cell granulomas, and the exclusion of known causes of granulomatous reactions [5, 6]. In fact, noncaseating granulomas are nonspecific for sarcoidosis and are associated with some infections (such as tuberculosis and histoplasmosis), occupational and environmental exposures (such as beryllium), autoimmune disorders (such as Wegener's granulomatosis), and malignancy (such as lymphoma and solid tumors) [1].

Some patients with sarcoidosis are not disabled by the illness and therefore do not require treatment [4]. In general, treatment is initiated when impairment of organ function is imminent. Oral prednisone at a dose of 20 to 40 mg daily is the recommended regimen. In the case of adequate response after 1 to 3 months, the prednisone dose should be tapered to 5 to 15 mg daily, with treatment planned for at least 6 additional months [4]. Sarcoidosis associated with massive splenomegaly can be treated with either splenectomy or corticosteroids, with no clear superiority of one modality over the other [2, 7]. Splenectomy has not been shown to alter the course of sarcoid progression. The indications for splenectomy include intractable abdominal pain from splenomegaly, functional asplenia, splenic rupture, hematologic abnormalities, massive splenomegaly refractory to medical therapy, or a strong suspicion of an alternative diagnosis [8].

Splenic involvement in sarcoidosis is defined as the histologic presence of noncaseating granulomas in the spleen. Autopsy studies show that the spleen is the second most commonly affected organ in sarcoidosis, with the lung being first [3]. Clinical evidence of splenomegaly is however uncommon, present only in up to 27% of cases. Moreover, the occurrence of massive splenomegaly in sarcoidosis is limited to case reports. Although there is no consensus regarding the definition of massive splenomegaly, most authors describe it as when the spleen reaches the pelvis or has crossed the midline into the right lower or right upper abdominal quadrants. Other authors define it as when the spleen weights more than 1000–1500 g or if the largest dimension is greater than 20 cm (Poulin et al.). The most common etiologies of massive splenomegaly include hematological disorders (such as myeloproliferative disease and lymphomas), infectious diseases (such as visceral leishmaniasis and malaria), and infiltrative conditions (such as Gaucher disease) [9]. Massive splenomegaly remains a rare manifestation of sarcoidosis. In fact, in a large review by Fordice et al. of 6074 cases of sarcoidosis, only 20 patients (3%) had massive splenomegaly [10]. Differential diagnosis of massive splenomegaly is as follows:

 Myelofibrosis (primary or secondary).
 Chronic myeloid leukemia.
 Lymphoma (usually indolent).
 Hairy cell leukemia.
 Gaucher disease.
 Amyloidosis.
 Beta thalassemia major.
 Schistosomiasis.
 Kala-azar (visceral leishmaniasis).
 Sarcoidosis (rarely).
 Hyperreactive malarial splenomegaly syndrome (tropical splenomegaly syndrome).
 AIDS with mycobacterium avium complex.
 Splenic vein thrombosis.

Splenic involvement in sarcoidosis is usually asymptomatic, although left upper quadrant pain is occasionally present. Patients with splenomegaly may have a higher incidence of constitutional symptoms and more disseminated disease [11]. Splenic sarcoidosis may cause hypersplenism, as evidenced by anemia, leukopenia, thrombocytopenia, or any such combination. The radiographic features of splenic sarcoidosis are variable. Splenomegaly is usually homogeneous; however, the sarcoid granulomas, often small, can coalesce to produce macroscopically visible nodules. Therefore, in up to 15% of patients, the disease may manifest as multiple low-attenuation and diffusely scattered nodules, ranging in size from 1 to 30 mm [2, 12, 13]. This pattern may mimic other worrisome diagnoses, such as lymphoma, metastases from solid tumor, and tuberculosis [2, 3].

The crux of sarcoidosis is its ability to masquerade as other diseases, most significantly lymphoma. As such, for clinicians, distinguishing these entities can make the difference between life and death for patients. In addition to the nonspecific clinical, radiological, and histological features in sarcoidosis, the lymphocyte activation and the reticuloendothelial system involvement (lymph nodes, spleen, and liver) make the distinction of sarcoidosis from lymphoma extremely challenging. In fact, hypercalcemia and increased serum ACE levels have also been described in patients with lymphoma [14]. Moreover, multiple reports confirmed the presence of sarcoid-like (noncaseating epithelioid) granulomas in patients with lymphoma, even without a history of “true” sarcoidosis [15]. Brincker concluded that a sarcoid-like granulomatous reaction occurred in 4% of cancers, in 14% of patients with Hodgkin's lymphoma, and in 7% of patients with non-Hodgkin's lymphoma [15]. In our patient, the combination of bicytopenia, hypercalcemia, diffuse lymphadenopathy, and massive splenomegaly favored a diagnosis of lymphoma. Moreover, the patient had no clinical or radiological evidence of respiratory system involvement to suggest sarcoidosis as a “likely” diagnosis.

In some cases, the coexistence of “true” sarcoidosis and lymphoproliferative disease has been reported in the literature. In most of these cases, sarcoidosis preceded the diagnosis of lymphoma, but in few other reports, lymphoproliferative disease occurred first [16]. This possible association between these two entities led to the so-called “sarcoidosis-lymphoma syndrome,” first suggested by Brincker in 1989 [17]. Since the diagnosis of sarcoidosis preceded the occurrence of the lymphoproliferative disease in most cases, he suggested that sarcoidosis might be a paraneoplastic syndrome [17]. The causal relation between these two entities is still a subject of speculation. It has been suggested that the impairment of the immune system in sarcoidosis, in the form of altered cell reaction and increased mitogenesis of B and T lymphocytes, can predispose to the development of lymphoid malignancies [18]. Moreover, the treatment of sarcoidosis with steroids can further compromise the immune system and may represent another predisposing factor for lymphoma development [15].

FDG PET imaging remains an essential modality in the management of lymphoma. One of many advantages it offers over conventional imaging is the ability to detect occult lesions. However, its specificity is limited by multiple false-positive conditions, including infections, inflammations, and sarcoidosis [19, 20]. In fact, in the setting of sarcoidosis, PET imaging has been suggested to monitor disease progression and response to therapy [21]. Since both sarcoidosis and lymphoma are FDG avid, PET imaging cannot differentiate these two conditions, and therefore histological verification remains mandatory [20, 22].

## 4. Conclusion

This report illustrates an unusual case of sarcoidosis that presented as bicytopenia, hypercalcemia, diffuse lymphadenopathy, and massive splenomegaly, mimicking lymphoma. Physicians should be aware of this atypical presentation and accordingly should consider sarcoidosis in their differential diagnosis, after excluding other worrisome diagnoses, such as lymphoma.

## Figures and Tables

**Table 1:** Laboratory findings.

Parameter	Value
Total protein (g/dL)	8.1 (6–8.3)
Albumin (g/dL)	3.2 (3.0–5.5)
Serum iron (*µ*g/dL)	34 (35–150)
Total iron binding capacity (*µ*g/dL)	241 (260–400)
Ferritin (ng/mL)	608 (30–400)
Percent saturation (%)	14.1 (15–50)
Reticulocyte count (%)	1.88 (0.5–1.5)
ESR (mm/h)	93 (0–10)
Vitamin B12 (pg/mL)	215 (243–894)
Lactate dehydrogenase (IU/L)	97 (60–200)
Inorganic phosphorus (mg/dL)	2.5 (2.1–4.9)
Intact PTH (pg/mL)	5 (15–65)
PTH related protein (pg/mL)	22 (14–27)
Thyroid stimulating hormone (*µ*IU/mL)	2.72 (0.27–4.2)
Vitamin D 1,25(OH)_2_ total (pg/mL)	248 (18–72)
Vitamin D 25-OH total (ng/mL)	26 (30–100)
Uric acid (mg/dL)	6.3 (4.8–8.7)
Serum protein electrophoresis (SPEP)	Normal
Free kappa/lambda ratio	1.01 (0.26–1.65)
Urine protein electrophoresis (UPEP)	Normal
